# Lignin-Stabilized Doxorubicin Microemulsions: Synthesis, Physical Characterization, and In Vitro Assessments

**DOI:** 10.3390/polym13040641

**Published:** 2021-02-21

**Authors:** Abbas Rahdar, Saman Sargazi, Mahmood Barani, Sheida Shahraki, Fakhara Sabir, M. Ali Aboudzadeh

**Affiliations:** 1 Department of Physics, University of Zabol, Zabol 98613-35856, Iran; 2 Cellular and Molecule Research Center, Resistant Tuberculosis Institute, Zahedan University of Medical Sciences, Zahedan 98167-43463, Iran; sgz.biomed@gmail.com (S.S.); sheida.shahraki@gmail.com (S.S.); 3 Department of Chemistry, Shahid Bahonar University of Kerman, Kerman 76169-14111, Iran; mahmoodbarani7@gmail.com; 4 Faculty of Pharmacy, Institute of Pharmaceutical Technology and Regulatory Affairs, University of Szeged, H-6720 Szeged, Hungary; fakhra.sabir@gmail.com; 5 Centro de Física de Materiales, CSIC-UPV/EHU, Paseo Manuel Lardizábal 5, 20018 Donostia-San Sebastián, Spain; 6 Donostia International Physics Center (DIPC), Paseo Manuel Lardizábal 4, 20018 Donostia-San Sebastián, Spain

**Keywords:** microemulsion, doxorubicin, *in vitro*, cytotoxicity, lignin (LGN)

## Abstract

Encapsulation of the chemotherapy agents within colloidal systems usually improves drug efficiency and decreases its toxicity. In this study, lignin (LGN) (the second most abundant biopolymer next to cellulose on earth) was employed to prepare novel doxorubicin (DOX)-loaded oil-in-water (O/W) microemulsions with the aim of enhancing the bioavailability of DOX. The droplet size of DOX-loaded microemulsion was obtained as ≈ 7.5 nm by dynamic light scattering (DLS) analysis. The entrapment efficiency (EE) % of LGN/DOX microemulsions was calculated to be about 82%. In addition, a slow and sustainable release rate of DOX (68%) was observed after 24 h for these microemulsions. The cytotoxic effects of standard DOX and LGN/DOX microemulsions on non-malignant (HUVEC) and malignant (MCF7 and C152) cell lines were assessed by application of a tetrazolium (MTT) colorimetric assay. Disruption of cell membrane integrity was investigated by measuring intracellular lactate dehydrogenase (LDH) leakage. *In vitro* experiments showed that LGN/DOX microemulsions induced noticeable morphological alterations and a greater cell-killing effect than standard DOX. Moreover, LGN/DOX microemulsions significantly disrupted the membrane integrity of C152 cells. These results demonstrate that encapsulation and slow release of DOX improved the cytotoxic efficacy of this anthracycline agent against cancer cells but did not improve its safety towards normal human cells. Overall, this study provides a scientific basis for future studies on the encapsulation efficiency of microemulsions as a promising drug carrier for overcoming pharmacokinetic limitations.

## 1. Introduction

Doxorubicin (DOX) is an anthracycline and active anticancer drug that contains a naphthacenequinone nucleus with a glycosidic bond to an amino sugar [1]. DOX is structurally similar to daunorubicin except that the former contains a hydroxyl acetyl group instead of an acetyl group at the 8-position. DOX is slightly soluble in normal saline and sparingly soluble in alcohol, and has broad-spectrum activity against neoplasms, lymphomas, solid tumors, and breast tumors [2]. It has been widely used as a first-line therapy in testicular, breast, and hepatocellular carcinoma. DOX has different biomedical applications in various chemotherapeutic regimens. It was previously applied in combination with bleomycin, dacarbazine, cyclophosphamide vincristine, and prednisone for the treatment of non-Hodgkin’s and Hodgkin’s lymphomas. Another application of DOX and cyclophosphamide can be used as adjuvant therapy with or without including fluorouracil followed by paclitaxel for breast cancer. The combination of DOX with a greater dose of cisplatin and methotrexate has been successfully applied to treat osteogenic sarcoma [3,4]. DOX attachment to DNA via intercalation could inhibit the function of topoisomerase II, resulting in disruption of DNA and RNA. The quinone group of DOX is reduced by cytochrome P450 reductase to produce semiquinone oxygen free radicals that can attack the cells’ DNA.

Moreover, DOX binds to cell membranes and modifies the fluidity of ion transport [5,6]. The cell membranes are permeable to the lipid-soluble anthracycline molecules with an unprotonated sugar amino group like DOX with a pKa value of about 8.2. These compounds have direct access to the intracellular sites in all cells, including tumor cells.

The limitations in the application of conventional DOX are its bone marrow suppression, nephrotoxicity, and cardiotoxicity. The toxicity issues related to DOX limit its long-term use for clinical purposes [7,8]. P-glycoprotein (P-gp) also shows multidrug-resistance-associated protein-1 (MRP1)-mediated efflux that makes the tumor less responsive towards DOX. The other challenges are its short half-life, poor solubility, lack of oral dosage formulations, instability of the drugs under gastric conditions, and hepatic first-pass effect [9,10]. Several approaches have been used to reduce the toxicity and enhance the oral efficacy of DOX. Some of them employ prolonged infusion along with simultaneous administration with other cardioprotective agents (dexrazoxane) [11]. Furthermore, anthracycline analogs and other novel drug delivery systems can be applied to modify its distribution and reduce its accumulation in the heart and lower its toxicity. DOX can be incorporated within different carrier systems, for example, DOX-loaded liposomes for efficient targeting against tumors [6]. Furthermore, encapsulation of DOX in dendrimers, nanocrystals, nanogels, nanotubes, and nanoemulsions has been studied [12].

Microemulsion, as a potential drug delivery system, allows controlled or sustained release of drugs for oral, topical, ocular, and percutaneous administration. In comparison to other dosage forms, microemulsions offer the advantages of easy formation, high scale-up, greater stability, the higher drug solubilization of hydrophobic drugs, and enhanced bioavailability [13,14]. A microemulsion system composed of oil, surfactant (non-ionic or anionic), co-surfactants, and water can form a large number of configurations and phases via mixing different proportions of components to design the formulation [14,15]. Non-ionic surfactants are preferable because of their high tolerability, lower irritation and toxicity, and they have the potential to enhance the biocompatibility of the colloidal system. Microemulsions can be developed by applying single or double chain surfactants. Single chains do not lower the interfacial tension, and that is why co-surfactants are needed. The co-surfactants may exhibit toxicity in the formulation of microemulsions [16]. In this context, the selection of surfactant and co-surfactant is of great significance. Phospholipids-based microemulsions are preferred over other synthetic surfactants from a toxicity point of view [17]. Green surfactants (GSs) provide benefits over other synthetic surfactants in many drug delivery methods [18]. The most relevant properties of GSs are biodegradability, economic production, environmental tolerance, specificity, and structural diversity [4,19,20]. Despite the many advantages of GSs such as the commercialization and economic availability in industries, they are not productive enough due to the high operational and material costs needed for their synthesis. Hence, the use of nanotechnological methods to increase the production of GSs via microbial induction has been evaluated. A lot of research studies have been carried out on decreasing the cost sources for manufacturing GSs [21]. 

Lignin (LGN) (a phenolic polymer derived from phenylpropanoid units,) is the type of GS that we used in this study. This biodegradable natural polymer, which is the result of industrial wood processing, has widespread implementations as a renewable bioresource for producing different end-products including detergents, surfactants, and dispersants [22]. LGNs are developed from different methods, including chemical modifications using alkylation, destruction, sulfonation, etc. [23]. LGN is also a potential material for biomedical applications because of its eco-friendly properties such as biodegradability, biocompatibility, and low toxicity [24,25]; due to its specific aromatic structure, it can be easily adsorbed at various interfaces. That is why among the applications proposed for lignin particles, interfacial stabilization is highly promising, especially for the encapsulation of sensitive drugs such as DOX. For example, Zhou et al. developed LGN-based hollow nanoparticles (NPs) to deliver DOX. The results showed that the folic magnetic functionalized LGN hollow NPs could enhance the cellular uptake of NPs into HeLa cells [26]. In another study, the same research group applied these LGN hollow NPs as useful vehicles for the antineoplastic drug DOX. The authors related the enhanced encapsulation efficiency and drug loading of the NPs to the surface area and pore volume. In this study, the focus was on the mechanism of encapsulation of DOX into LGN hollow NP. The results of loading and release behavior of DOX were due to the various structures, stabilities, and sizes of LGN hollow NPs. The controlled release behavior of LGN hollow NPs, the access of DOX-loaded LGN hollow NPs into cells, and the low pH environment of lysosomes can support the release of DOX [27]. Whereas these pioneering reports made use of complex nanocarriers such as hollow NPs, here, we report the facile encapsulation of DOX into microemulsion systems which are more stable, easy-to-formulate, along with the greater potential for industrial scale-up. This novel strategy formulates a novel green carrier (DOX-loaded microemulsion) that has not been implemented before for these types of cancer cell lines (MCF7 and C152). In addition, in this formulation, we used a low amount of surfactant for the preparation of microemulsions, which can overcome the challenges and issues related to the toxicity of synthetic surfactants. These findings may be further sculpted into new cancer treatment strategies if this newly synthesized formulation induces desirable cytotoxic activity.

## 2. Materials and Methods

### 2.1. Chemicals

Standard laboratory-grade chemicals, including DOX, lignin (alkali) with CAS 8068-05-1, sodium caprylate, and ethyl butyrate, were provided by Sigma Chemical Co (St. Louis, MI, USA). Fetal bovine serum (FBS) and culture media (RPMI1640 and Dulbecco’s modified Eagle’s medium (DMEM)) were all obtained from GIBCO (Grand Island, NY, USA). Dimethyl sulfoxide (DMSO), 1% penicillin/streptomycin solution, and phosphate-buffered saline (PBS) were purchased from KalaZist company (KalaZist Co., Tehran, Iran). Amphotericin B and 3-(4,5-Dimethylthiazol-2-yl)-2,5 diphenyltetrazolium bromide (MTT) were procured from Sigma-Aldrich Co. (St. Louis, MI, USA). All plastic materials were provided by Sorfa Medical Plastic Co. (Hangzhou, China).

### 2.2. Cell Lines 

Human oral squamous carcinoma (C152) and human umbilical vein endothelial (HUVEC) cell lines were obtained from the cell bank of Pasteur Institute of Iran (Tehran, Iran) and cultured in DMEM medium. Michigan Cancer Foundation-7 (MCF7) human breast cancer cells were obtained from the cell repository of the Research Institute of Biotechnology, Ferdowsi University of Mashhad (Mashhad, Iran) and maintained in RPMI1640 medium. MCF7 and C152 were selected as appropriate *in vitro* models for solid tumors. Both cell lines are well-suited for *in vitro* cytotoxic assessments and morphology evaluations since they can be cultured easily. HUVEC was chosen as a widely studied non-cancerous cell line for further assessments. Culture media were supplemented with 250 µg/mL of amphotericin B, 50 U/mL of penicillin, 50 µg/mL of streptomycin, and 10% heat deactivated FBS. Cells were maintained under standard culture conditions, described in our previous work [28]. 

### 2.3. Formulation of DOX-Loaded Microemulsions

**Scheme 1:**
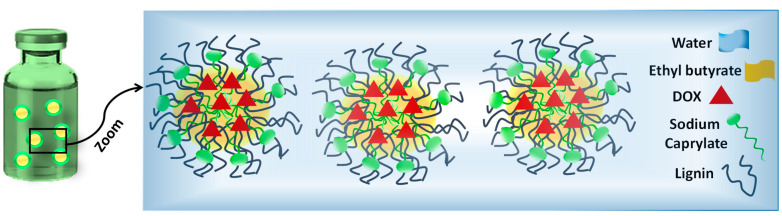
Schematic representation of DOX-loaded O/W microemulsion and its contents prepared in this study.

DOX-loaded O/W microemulsions were prepared according to a reported procedure [29], as 1% *w/w* solutions of oil by dissolving appropriate quantities of LGN, fatty acid (sodium caprylate), and finally, ethyl butyrate in PBS (pH 7.4) via vigorous stirring at an oil-to-LGN molar ratio of 1. All the preparation steps were carried out in a room-temperature environment. The microemulsions were subsequently allowed to equilibrate for at least 1 day prior to use. A schematic depiction of the contents of the microemulsions prepared in this work is shown in Scheme 1.

### 2.4. Characterization of DOX-Loaded Microemulsions by DLS

DLS characterization of DOX-loaded LNG microemulsions was carried out using an ALV-5000F Goniometer System (Sartorius, Germany) coupled with a diode-pumped solid-state laser. These measurements were performed at an angle of θ = 90° to the incident ray through calibrating the intensity scale by toluene against scattering. The system was also integrated with a digital correlator (ALV SP-86, Sartorius, Germany) with a sample range of 25 ns to 100 ms. A description of DLS and the mechanism of data analysis via this technique are presented in the Supporting Information. The polydispersity index was calculated by the average size of the droplets divided by the average number of measured droplets.

### 2.5. Entrapment Efficiency of DOX

To study the entrapment efficiency (EE), DOX was loaded into LGN-stabilized microemulsions, and the percentage of EE was determined by a UV spectrophotometric approach (Agilent Technologies, Cary 50, Pittsburgh, USA) [30,31,32]. Stock solutions of DOX with a concentration of 2 to 250 μg/mL were prepared with ethanol/PBS 7.4 (1:1). The absorbance peak of DOX was recorded in the range 250 to 750 nm, where DOX showed a characteristic peak at a wavelength of 480 nm. Based on this characteristic peak, the calibration curve of standard DOX was achieved with an R^2^ of 0.9894. LGN/DOX were centrifuged at 20,000 rpm for 60 min (model MC-20,000, Medline, UK). The centrifuging procedure was continued until a clear supernatant was obtained. The absorbance of the supernatant was measured at 480, and the EE of the DOX in the LGN/DOX was determined using Equation (1).

(1) $$\mathrm{Entrapment}\;\mathrm{effeciency}\%=\frac{\left(\mathrm{Total}\;\mathrm{Dox}-\mathrm{Free}\;\mathrm{Dox}\right)}{\mathrm{Total}\;\mathrm{Dox}}\times 100$$

### 2.6. Release Study

The release rate was evaluated using the dialysis technique with a 6000 Dalton pore size dialysis membrane [30,31,33]. First, the dialysis bags were immersed in the PBS buffer for 24 h (24 h). Then, 1 mL of standard DOX solution or LGN/DOX microemulsion was placed in the dialysis bag and immersed in 50 mL PBS 7.4: ethanol (in 1:1 ratio as the buffer solution). The dialysis bag was left to stir at 90 rpm at 37 °C using a heater stirrer. At different time intervals up to 24 h, 1 mL of the buffer solution was removed and then replaced by adding the same volume of fresh buffer (preheated at 37 °C). The UV spectrophotometer recorded the absorbance of the samples at 480 nm to quantify released DOX.

To investigate the nature of the release mechanism, based on our previous studies, we have fitted the *in vitro* DOX release with different kinetic methods [30,31,32,34]. 

### 2.7. Cell Viability Assay and Evaluation of Cell Morphology

The target cells used in this experiment were C152 and MCF7, the two well-studied cell lines for analyzing the *in vitro* growth-inhibitory effects of anticancer agents. Endothelial HUVEC cells were used as target non-malignant cells. The cytotoxic effects of standard DOX and LGN/DOX microemulsions were evaluated using the MTT reduction assay [35].

Cells (5×10^3^ cell/well) were seeded in 96-well microplates and incubated for 24 h to obtain a monolayer culture. Next, cells were treated with increasing DOX concentrations in standard or encapsulated state from 0.0625 to 1 µg/mL, and incubated for 48 h. Then, the supernatant was removed from each well, and 200 μL MTT reagent (5 mg/mL) was placed into each well and kept for another 3 h in a humidified incubator. The MTT solution was then replaced with 200 μL of DMSO, and the plates were placed on a shaker to dissolve the formazan crystals completely. The optical density (OD) of each well was measured at a test wavelength of 570 nm using a microplate reader (Stat Fax 2100, Awareness, Technologies Inc, Palm City, FL, USA). The percentage of viable cells was calculated using the following formula:

Cell viability (%) = OD sample/OD control × 100(2)

The half-maximal inhibitory concentration (IC50) of LGN/DOX microemulsions and standard DOX was calculated via GraphPad Prism software version 7.0.

For morphology assessment, cells were plated in 6-well microplates at a density of 1 × 10^5^ cell/well. Following overnight incubation, cells were treated with given MTT concentrations for 48h, and untreated cells served as controls. Changes in cell morphology were monitored by an inverted phase-contrast microscope (IX71, Olympus Inc., Japan) at 20× magnification and imaged using a digital camera.

### 2.8. Membrane Integrity

Lactic dehydrogenase (LDH) leakage was measured in the medium of cultivated cells using a cytotoxicity assay kit (Cayman item no 601170) (Cayman Chemical Company, Ann Arbor, MI, USA) to evaluate membrane integrity. Cells were inserted in 96-well microplates (5 × 10^3^ cell/well) and kept in an incubator for 24 h. Next, the culture medium was removed, and cells were exposed to a concentration equal to IC50 concentrations of both agents (standard DOX and LGN/DOX microemulsions). After treatment for 48 h, 100 µL of supernatant was placed into each well of a 96-well microplate for assessment. The LDH leakage (% of total) as a marker of cytotoxicity was determined as percentage of LDH in medium compared with total LDH in cell lysate for each microwell using the following formula:

LDH leakage (%) = (OD_test_ − OD_spontaneous release_)/(OD_maximum release_ − OD_spontaneous release_)×100 (3)

where OD_test_ is the absorbance of cells treated with our microemulsion, OD_spontaneous release_ is the absorbance of microwells containing assay buffer, and OD_maximum release_ represents the absorbance of microwells containing cells.

Absorbance was read at 490 nm via a microplate reader (Spectra Max Gemini ®, Molecular Devices Cooperation, Sunnyvale, CA, USA).

### 2.9. Statistical Analysis

Results were analyzed using SPSS software version 20 (SPSS Inc., Chicago, IL, USA). Differences among treated and untreated cells were determined using the sample T-test and non-parametric one-way analysis of variance (ANOVA) test. *p* ˂ 0.05 was considered statistically significant.

## 3. Results

### 3.1. Characterization of DOX-Loaded Microemulsions by DLS

Figure S1 (in supplementary materials) shows an autocorrelation function (ACF) against time for the prepared microemulsions. Fitting a curve to the ACF yielded a decay rate from which the droplet size of DOX-based microemulsions was measured to be approximately 7.4 nm based on the diffusion data (3.1938 × 10^−11^ m^2^/s) [30,31,32]. For encapsulating drugs, without any doubt, a small particle would be an advantage since it would carry only a small drug quantity, which will help in minimizing the onset of abrupt toxic response [36]. The PDI obtained from DLS analysis showed values (0.1–0.2), indicating a good size distribution of the oil droplet in the microemulsion system. This parameter directly reflects the size homogeneity of the droplets in the total microemulsion. The stability of the microemulsion was confirmed visually after passing three months of preparation and no aggregation was observed (Figure S1b).

### 3.2. Entrapment Efficiency

Generally, an ideal drug carrier should have high encapsulation efficiency (EE) [33,37,38,39]. High EE (above 70%) can increase the efficacy of the drug delivery system and decrease the side effects of the drug [33,39]. The EE% of the LGN/DOX microemulsion prepared in this study was calculated to be about 82%. Interactions between DOX and LGN microemulsions can lead to this high EE. LGN molecules have a similar polyphenolic structure to DOX, thus hydrogen bonding and π–π stacking enhance this interaction between them as well. The microemulsion core is hydrophobic, and it could be concluded that DOX was mainly entrapped in the core of the microemulsion. Moreover, the EE of our LGN/DOX microemulsion system is well above the EE of LGN-based NPs that have been designed for encapsulating different compounds [40]. For example, the EE of the pesticide avermectin (AVM) in hollow lignin azo colloidal spheres was determined to be 61.49% (*w/w*) [41]. In another similar study, Li et al. synthesized lignosulfonate-based colloidal spheres from a mixture of sodium lignosulfonate and cetyltrimethylammonium bromide through self-assembly for the encapsulation of AVM, and the EE of AVM reached up to 62.58% [42]. In contrast, when (similar to our study) DOX was used as the active compound, its encapsulation efficiency in LGN-based hollow nanoparticles (NPs) was obtained as 67.5% [26].

### 3.3. In Vitro Release Experiment

**Figure 1:**
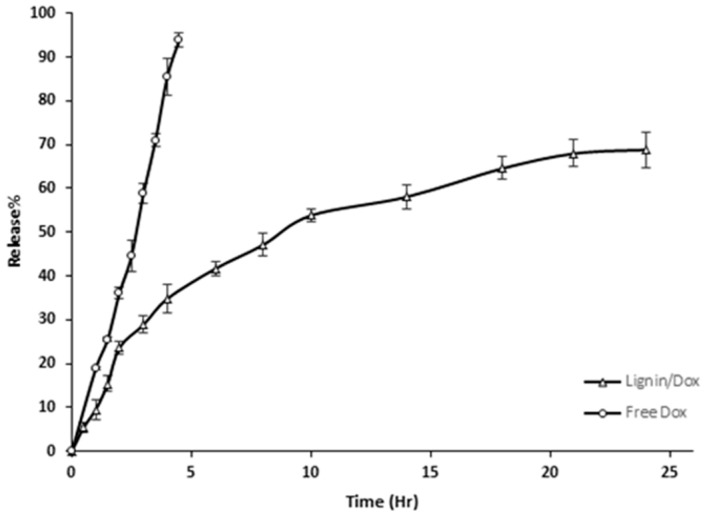
*In vitro* release behavior of free DOX and LGN/DOX microemulsions at PBS 7.4/ethanol (1:1) and 37 °C after 24 h. The error bars indicate the uncertainty.

One of the crucial characteristics of drug delivery systems in biomedicine is to impart the sustained release of a drug. Thus, the sustained drug release improves the accumulation of DOX at the tumor site while enhancing its anticancer efficiency. The *in vitro* release of DOX solution (standard DOX) and LGN/DOX microemulsions was performed using dialysis methods at PBS 7.4/ethanol (1:1) and 37 °C up to 24 h. As shown in Figure 1, the release rate of standard DOX was significantly faster than LGN/DOX microemulsions. The DOX release reached 68% after 24 h for the LGN/DOX microemulsion that showed a slow and sustainable release rate. 

In a similar study, Heggannavar et al. prepared DOX-loaded magnetic silica-pluronic F-127 nanocarriers and conjugated them with transferrin (Tf) to treat glioblastoma. The release of DOX in these thermosensitive NPs was predominant during the first 24 h at 37 °C and reached around 17.2%. However, when the temperature was raised to 42 °C, DOX release was significantly increased from 17.2 to 32.2% for 24 h and reached up to 51.4% for 120 h [43].

After matching the first-order, zero-order, Higuchi, and Korsmeyer–Peppas models with the DOX release profile, it was found that the kinetics of release followed the Higuchi model with an R^2^ of 0.965 (Figure S2 in supplementary materials). This describes that DOX release as a diffusion process is based on Fick’s law, which is square root time-dependent [30].

The effect of different parameters on the release of active ingredients from nanocarriers has been evaluated in several studies [37,38,39]. Thus, the release of DOX from the NPs was found to follow an anomalous type of behavior. For example, Heggannavar et al. investigated the nature of the DOX release mechanism and obtained values of n (a parameter that represents the nature of the release mechanism) varying from 0.6175 to 0.6589 [43]. Figure S2 shows profiles of different kinetic models for release of DOX from LGN/DOX microemulsions.

### 3.4. Effects of *Standard and Encapsulated DOX* on Cells Viability and Morphology

**Figure 2:**
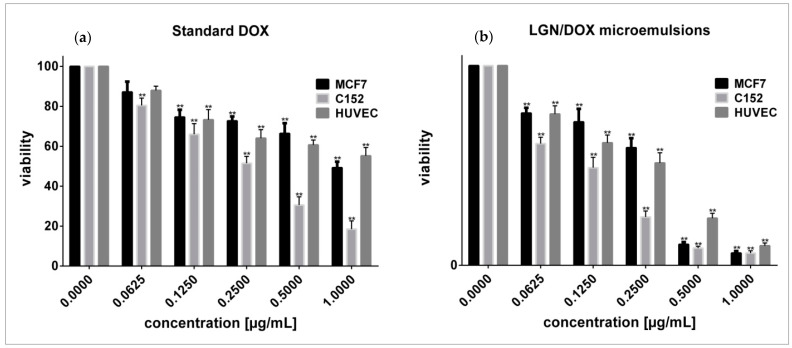
Cytotoxicity assessment of (**a**) standard DOX and (**b**) LGN/DOX microemulsions on malignant (MCF7, C152) and non-malignant (HUVEC) cells following 48 h treatment. (** *p* < 0.05 compared with untreated cells).

Compared with untreated cells, incubation with different concentrations of standard DOX significantly inhibited the proliferation of MCF7, C152, and HUVEC cells in a dose-dependent fashion (Figure 2). Likewise, exposing these cells to gradually increasing concentrations of LGN/DOX microemulsions exhibited a dose-dependent inhibitory effect and a significant reduction in the number of MCF7, C152, and HUVEC cells compared with untreated cells (*p* < 0.05), presenting cell viability that ranged from 6.14 to 76.23% (for MCF7 cells), 6.16 to 60.95% (for C152 cells), and 9.82 to 75.78% (for HUVEC cells) (Figure 2). IC50 values for 48 h treatment of MCF7, C152, and HUVEC cells with standard DOX were 0.798, 0.244, and 0.716 μg/mL, respectively. At the same time, the IC50 concentrations of LGN/DOX microemulsions were 0.197 μg/mL (for HUVEC cells), 0.094 μg/mL (for C152 cells), and 0.208 μg/mL (for MCF7 cells). Between the studied cell lines, C152 cells were most susceptible to encapsulated DOX. Our MTT assay results showed that encapsulated DOX induced higher toxicity than standard DOX on normal HUVEC cells, indicated by a lower IC50. 

**Figure 3:**
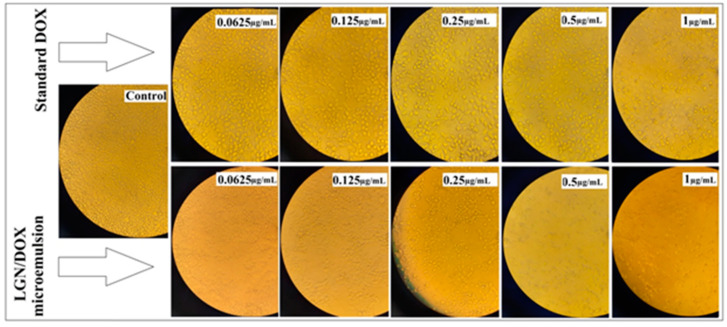
Optical microscopy images of malignant C152 cells treated with standard DOX and LGN/DOX microemulsions for 48 h.

**Figure 4:**
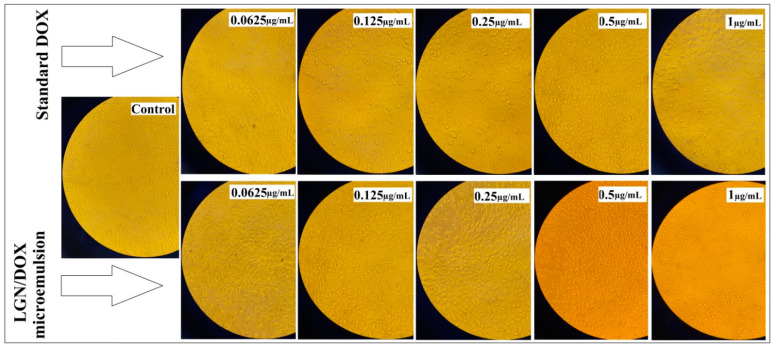
Optical microscopy images of malignant MCF7 cells treated with standard DOX and LGN/DOX microemulsions for 48 h.

**Figure 5:**
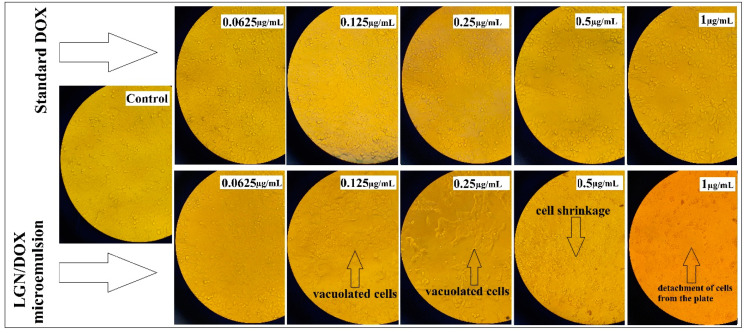
Optical microscopy images of non-malignant HUVEC cells treated with standard DOX and LGN/DOX microemulsions for 48 h.

All three cell lines were characterized by altered morphology and proliferation rate. MCF7 cells grew faster, and the cells were larger and more branched than the other two cell lines. Among the studied cell lines, the HUVEC cells were relatively smaller and were more compact. Gradual increasing concentrations of standard DOX could moderately decrease the viability of C152 (Figure 3), MCF7 (Figure 4), and HUVEC (Figure 5) cells without causing noticeable morphologic changes. Contrastingly, treatment with LGN/DOX microemulsions caused evident concentration-dependent morphological changes, specifically in C152 cells (Figure 3). In this regard, treatment with 0.0625 µg/mL nano-DOX decreased the number of living C152 cells, while higher concentrations of nano-DOX caused progressive nuclear shrinkage and formation of apoptotic bodies. Upon exposing MCF7 cells to nano-DOX at a concentration of 0.5 µg/mL, cells were shrunk, and apoptotic bodies were similarly formed (Figure 3). The highest concentration of LGN/DOX microemulsions (1 µg/mL) led to the adhesion of MCF7, C152, and HUVEC cells to the plate, which was not observed when cells were treated with standard DOX at given concentrations (Figure 3, Figure 4, and Figure 5).

The clinical success of nanomedicine relies on the synthesis of stable nanocarriers that can expeditiously encapsulate chemotherapeutics and quickly release them into target malignant cells. Anthracyclines entrapped in small-sized nanocarriers have the advantage of reduced systematic toxicity and gradual release of the payload [44]. In the current study, we observed that LGN/DOX microemulsions showed higher cytotoxic activity compared to standard DOX, indicating that the different level of cytotoxic activity is due to the encapsulation of DOX. It has been demonstrated that the encapsulation process boosts the therapeutic efficacy and the intracellular uptake of DOX [45]. The incorporation of DOX into long-circulating liposomes resulted in increased DOX accumulation in tumor tissue associated with higher toxicity than unencapsulated DOX [46]. 

Lately, technological advancements have been made in controlling the release of anticancer drugs and enhancing their solubility and stability by applying innovative drug delivery systems, such as microemulsions [47]. Multiple surfactant-based delivery systems, such as microemulsions, have recently been designed for efficient localization or systematic delivery of antitumor drugs to tumor cells [48]. Shakeel and coworkers showed that 5-fluorouracil (5-FU) in an optimized nanoemulsion inhibits the proliferation of SK-MEL-5 tumor cells more effectively than free 5-FU [49]. Chen et al. showed how Pluronic-based functional polymer mixed nanomicelles could serve as a potential nanodrug delivery system for efficient co-delivery of DOX and paclitaxel, two conventional chemotherapeutic agents [50]. In our study, the enhanced antitumor activity of LGN/DOX microemulsions might be due to the prolonged and controlled release of DOX by using this new formulation.

Similarly, few studies reported the desirable antitumor efficacy of DOX-loaded nanocarriers. In 2019, Abbasian et al. investigated the growth-inhibitory effects of newly designed LA/chitosan/NaX/Fe_3_O_4_/DOX nanofibers. They observed that these nanofibers decreased the number of viable H1355 cancer cells by 82% following a seven-day treatment [51]. Hence, as a beneficial carrier for hydrophobic antitumor agents, LGN-based microemulsions can be applied to treat solid tumors.

On the other hand, alkylating agents may cause adverse effects on normal cells; therefore, their application for chemotherapy is limited [52]. Compared with standard DOX treatment, we noticed a lower IC50 concentration for nano-DOX treated cells derived from human umbilical vein endothelial cells. This might not be a desirable effect for our newly developed encapsulated DOX since these microemulsions were cytotoxic at high concentrations toward normal human cells. More studies are needed to fully elucidate the cytotoxic activity of LGN-based microemulsions on normal cells derived from different human tissues.

### 3.5. LDH-Based Cytotoxicity Assay 

**Figure 6:**
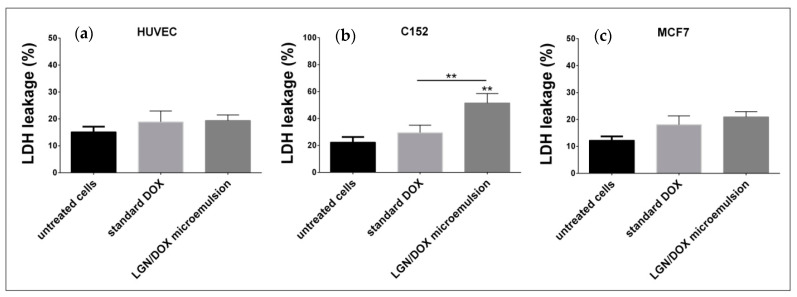
Effect of standard DOX and LGN/DOX microemulsions on the membrane integrity of (**a**) HUVEC, (**b**) C152, (**c**) MCF7, and cells after 48 h of exposure. ** *p* < 0.05.

If the cell membrane is disrupted, intracellular LDH is released from the cytosol into the culture medium. Therefore, the percentage of LDH leakage is an indicator of cell membrane damage as a result of necrosis or late-stage apoptosis. In comparison to control cells, exposure to standard DOX and LGN/DOX microemulsions enhanced LDH leakage by 1.48- and 1.71- (for MCF7 cells), 1.32- and 2.31- (for C152 cells), and 1.24- and 1.28- (for HUVEC cells) fold, respectively (Figure 6). Interestingly, compared to standard DOX, a significant increase in LDH leakage was observed following treatment of C152 cells with LGN/DOX microemulsions (*p* < 0.05). This shows that encapsulated DOX induced greater damage on cell membrane and might trigger necrosis as an alternative cell death mechanism. Compared to standard DOX, LGN/DOX microemulsions did not augment the leakage of cytosolic LDH in HUVEC and MCF7 cells (*p* > 0.05).

Former studies demonstrated that apoptotic cell death could be the principal mechanism of DOX-induced cell death [53]. Recently, it has been shown that Bcl-2-like 19kDa-interacting protein 3 [54] and poly-ADP-ribose polymerase 1 [55] could mediate DOX-induced necrosis as well. In the current study, cells were treated with standard DOX and LGN/DOX microemulsions, and some hallmarks of apoptotic cell death, including the formation of membrane-bound apoptotic bodies, cell shrinkage, and roundness, appeared as evident morphological alterations. On the other hand, loss of membrane integrity and LDH leakage are indications of necrosis [56], a type of cell-death that lacks features of apoptosis and is generally considered to be uncontrolled [57]. In our study, changes in LDH leakage were different for the investigated cell lines. In comparison to untreated cells, encapsulated DOX did not enhance LDH leakage in HUVEC cells, which is a promising outcome. In contrast, both agents did not significantly enhance LDH leakage in malignant MCF7 cells, recommending that apoptosis might be the primary mechanism of cell death in these cells. This is in agreement with the findings of Pilco-Ferreto et al., suggesting that DOX activating apoptosis in MCF7 cells is the main mechanism of cell death through inducing proteolytic processing of anti-apoptotic proteins, i.e., B cell lymphoma 2 (BCL2) family members [58]. Additionally, Sharifi and coworkers confirmed that DOX induces mitochondrial-dependent apoptosis by up-regulating *caspase-9* and proapoptotic protein (*Bax*), and down-regulating an anti-apoptotic protein (*Bcl-xL*) [59]. However, compared to untreated cells, we observed enhanced LDH leakage like treated C152 cells with encapsulated DOX. To the best of our knowledge, no study has already established the principal mechanism for DOX-induced cell death in C152 oral carcinoma cells. Another study also reported that DOX treatment causes necrotic cell death, mostly in cells that had floated from culture plates [60]. This suggests that two modes of cell death might be induced by DOX in different cell lines. Thus, changes in LDH leakage between MCF7 and C152 cells might be due to the activation of distinct cell death pathways within these cells. Furthermore, LGN/DOX microemulsions induced more toxicity in C152 cells compared with standard DOX. This might explain the significant enhancement in LDH leakage in C152 cells exposed to this formulation.

Overall, these findings suggest that LGN/DOX microemulsions might induce cell death through activation of both apoptosis and necrosis mechanisms. Further studies are needed to discover the underlying cell death mechanisms by which these microemulsions kill cancer cells. 

### 3.6. Practical Applications and Future Research Perspectives

Novel methods for preparing drug-loaded microemulsion systems and further investigation with the aim of expanding their practical applications are worth exploration. However, in drug delivery technology, employing these systems is limited because of their drug loading capacity and the used level of excipients [61]. In this context, surfactants and co-surfactants can be toxic at high concentrations and may be limited in their uptake levels. In the present study, we aimed to maximize DOX loading capacity while using the minimum amount of surfactant for preparing LGN/DOX microemulsions. A substantial amount of work on physicochemical O/W formulations and *in vitro* assessments are required to be performed before they can live up to their potential as widely used drug carriers. 

The diversity in lignin structure, chemical reactivity, and its safety profile can be further exploited in different applications of lignin-based materials. Advances in genetics, bio-, and analytical chemistry have resulted in a deeper understanding of lignin biosynthesis and the structure of nanoparticles in the field of nanomedicines.

## 4. Conclusions

Our results indicated that novel synthesized LGN/DOX microemulsions exert cytotoxic effects on oral and breast carcinoma cells while inducing unfavorable toxic effects on normal human cells. Performing more studies is encouraged to investigate the growth-inhibitory activity of this novel formulation on other malignant and non-malignant cell lines. Due to the toxic effects of LGN/DOX microemulsions on normal human cells, multiple considerations should be undertaken before using these microemulsions to treat cancer patients. The take-home message of the current research is that DOX encapsulation in O/W microemulsions enhanced its therapeutic efficacy against solid tumors. This could be further sculpted into new approaches for treating different types of cancers.

## Data Availability

The data presented in this study are available on request from the corresponding author.

## Supplementary Materials

The following are available online at https://www.mdpi.com/2073-4360/13/4/641/s1, Figure S1: Dynamic light scattering autocorrelation function of microemulsions Figure S2: Profiles of different kinetic models for release of DOX from LGN/DOX microemulsions.
