# Supplementary material for: Lignin-Stabilized Doxorubicin Microemulsions: Synthesis, Physical Characterization, and In Vitro Assessments
Source: Polymers (Basel). 2021 Feb 21;13(4):641. doi: 10.3390/polym13040641 (PMC7926373; doi:10.3390/polym13040641)
Supplement: Supplementary file 1 [file polymers-13-00641-s001.pdf]

polymers

Lignin-Stabilized Doxorubicin Microemulsions: Synthesis, Physical Characterization, and In Vitro Assessments

Abbas Rahdar [1,*], Saman Sargazi [2], Mahmood Barani [3], Sheida Shahraki [2], Fakhara Sabir [4] and M. Ali Aboudzadeh [5,6*]

[1] Department of Physics, University of Zabol, 98613-35856 Zabol, Iran
[2] Cellular and molecule Research Center, Resistant Tuberculosis Institute, Zahedan University of Medical Sciences, 98167-43463 Zahedan, Iran, sgz.biomed@gmail.com (S.S.), sheida.shahraki@gmail.com (S.S.)
[3] Department of Chemistry, Shahid Bahonar University of Kerman, 76169-14111 Kerman, Iran, mahmood-barani7@gmail.com
[4] Faculty of Pharmacy, Institute of Pharmaceutical Technology and Regulatory Affairs, University of Szeged, H-6720 Szeged, Hungary, fakhra.sabir@gmail.com
[5] Centro de Física de Materiales, CSIC-UPV/EHU, Paseo Manuel Lardizábal 5, 20018 Donostia-San Sebastián, Spain
[6] Donostia International Physics Center (DIPC), Paseo Manuel Lardizábal 4, 20018 Donostia-San Sebastián, Spain
* Correspondence: a.rahdar@uoz.ac.ir (A.R.); mohammadali.aboudzadeh@ehu.eus (M.A.A.)

Description of dynamic light scattering (DLS) technique and mechanism of data analysis

DLS is a powerful characterization tool to measure the particle size and diffusion coefficient of nanoparticles in a colloidal suspension. The time-mediated light scattering intensity from the colloidal suspension is a variable quantity dependent on Brownian motion, diameter, and the diffusive behavior of nanoparticles in the solution. These fluctuations can be characterized according to the normalized autocorrelation function, $g^1(\tau)$, of the dispersed electric field for a specified time, τ, which comprises information about the dynamics and structure Equation (1) [1–4].

$$g^1(q,\tau) = \frac{\langle E(q,t)\, E^*(q,t+\tau) \rangle}{\langle I(q,t) \rangle} \tag{1}$$

where, E^* denotes the complex conjugate of E. The intensity autocorrelation function, $g^2(q, \tau)$, is experimentally measured using the relation as follows Equation (2) [2–4]:

$$g^2(q,\tau) = \frac{\langle E(q,t)\, E^*(q,t) E(q,t+\tau) E^*(q,t+\tau) \rangle}{\langle I^2(q,t) \rangle} \tag{2}$$

The $g^2(q,\tau)$ is changed to $g^1(q,\tau)$, the autocorrelation function of the scattered electric field by the Siegert relationship Equation (3) [2–4].

$$g^2 \quad q, \qquad |A \exp(\quad \Gamma \quad)|^2 \tag{3}$$

For a colloidal system having colloidal particles such as micelles and other nanoparticles, a single exponential decay curve represents the function of $g^1(q, \tau)$ Equation (4) [2–4].

$$g^1 \quad q, \qquad A \exp(\quad \Gamma \quad) \tag{4}$$

It is important to note that there is a digital correlator within the DLS tool that assesses the extent of similarity between two signals over a period. If the signal's intensity of a specific part of the speckle pattern at a given point is comparable to the signal's strength a very short time later, the resulting two signals are very similar, and they

powerfully correlate with each other. On the other hand, the correlation between two signals decreases with time due to a decrease in the similarity of two signals by Brownian motion.

The decay rate, Γ, is transferred to the diffusion coefficient using the following equation Equation (5) [2–4]:

$$D = \Gamma/q^2 \tag{5}$$

where q illustrates the scattering vector [2–4]. The diffusion coefficient of nanoparticles or micelles can be characterized as R_h according to the Stokes–Einstein equation Equation (6) [2–4]:

$$R_h = \frac{k_B T}{6\eta\pi D} \tag{6}$$

An increase in particle sizes results in a slow exponential relax with a small relax constant value, as the fluctuations in light intensity change more slowly. In contrast, a stretched relaxing exponential function is obtained for smaller particles with a larger relaxing constant value. Thus, the inverse correlation time is inversely associated with the NPs' diameter/size [2–4].

(**a**) (**b**)

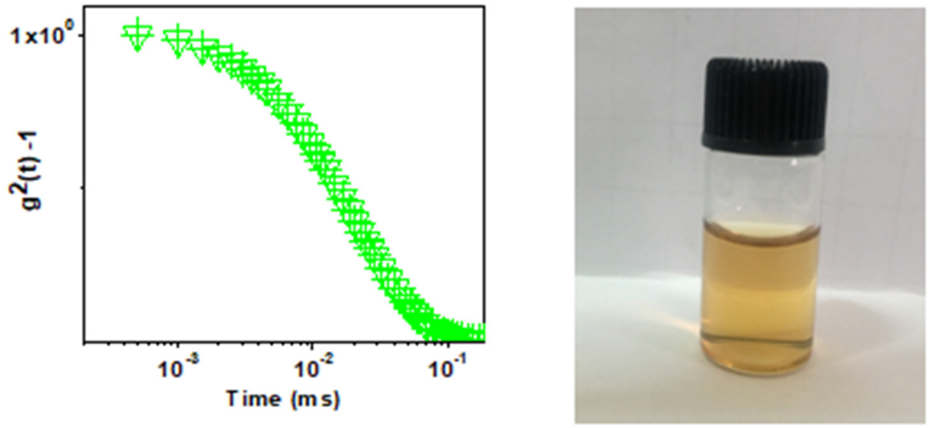

Figure S1. Dynamic light scattering autocorrelation function of microemulsions (**a**) and visual stability of microemulsion after 3 months (**b**).

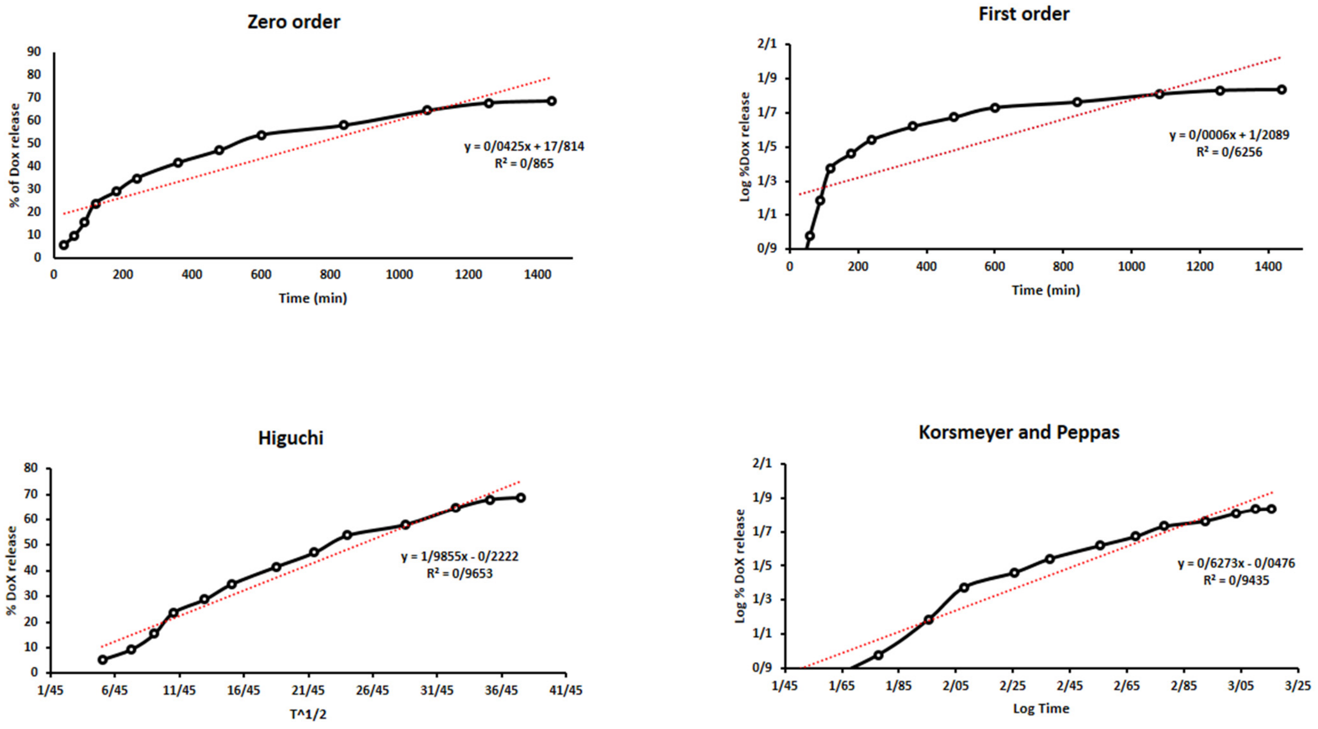

Figure S2. Profiles of different kinetic models for release of DOX from LGN/DOX microemulsions.

References:

1 Rahdar, A.; Almasi-Kashi, M.; Khan, A.M.; Aliahmad, M.; Salimi, A.; Guettari, M.; Kohne, H.E.G. Effect of ion exchange in NaAOT surfactant on droplet size and location of dye within Rhodamine B (RhB)-containing microemulsion at low dye concentration, *J. Mol. Liq.* **2018**, *252*, 506–513.

2 Rahdar, A.; Amini, N.; Askari, F.; Susan, M.; Hasan, A.B. Dynamic light scattering and zeta potential measurements: effective techniques to characterize therapeutic nanoparticles, *J. Nanoanalysis*, **2019**. Doi: 10.22034/JNA.2019.575819.1108

3 Brown, W. Dynamic light scattering: the method and some applications, Oxford University Press, New York, NY, USA, 1993.

4 Finsy, R. Particle sizing by quasi-elastic light scattering, *Adv. colloid interface Sci.* **1994**, *52*, 79–143.
